# Introducing the Including Disability in Education in Africa Research Unit at the University of Cape Town

**DOI:** 10.4102/ajod.v11i0.946

**Published:** 2022-01-24

**Authors:** Richard Vergunst, Judith McKenzie

**Affiliations:** 1 Department of Health and Rehabilitation Sciences, Faculty of Health Sciences, University of Cape Town, Cape Town, South Africa

**Keywords:** disability, education, research, Africa, unit

## Abstract

**Background:**

The Including Disability in Education in Africa (IDEA) Research Unit at the University of Cape Town in South Africa was established in 2020 and focused on carrying out research studies in the field of disability and education in Africa and beyond.

**Objectives:**

The objective of this article was to introduce the research unit and highlight its vision, mission and objectives.

**Method:**

A general review of the research unit.

**Results:**

The IDEA Research Unit plays an important role in the research arena within South Africa and Africa when it comes to disability issues in education.

**Conclusion:**

More networking and collaboration should take place between the IDEA Research Unit and relevant stakeholders in the field of disability and education.

## Background

There is a global move towards inclusive education (Donohue & Bornman 2015). Inclusive education entails identifying and removing barriers and providing reasonable accommodation in order to enable every child to participate and achieve within mainstream educational settings (United Nations 2016; World Health Organization 2011). The movement towards inclusive education has its roots in the Salamanca Statement (UNESCO 1994) through the United Nations Convention on the Rights of Persons with Disability (United Nations 2006) and most recently within the Sustainable Development Goals (SDGs) (United Nations Department of Economic and Social Affairs 2015). The adoption of the SDGs focused on the education of children and young people with disabilities, particularly in the global South (Taneja-Johansson, Singal & Samson 2021). Within the South African context, the constitution (Republic of South Africa 1996) and the *South African Schools’ Act* (Republic of South Africa 1996) affirm the right to education for all and Education White Paper 6: Special Needs Education – Building an Inclusive Education and Training System (Department of Education [DoE] 2001) provides specific policy and implementation strategies and targets for the inclusion of those who experience barriers to learning, including children with disabilities.

However, despite this context and education being recognised as a key issue for people with disabilities (Parnes et al. 2009), disability, in reality, remains a significant factor in exclusion from education and schooling that is evident in educational policy and practice (Bines & Lei 2011). This is particularly true for the global south where the impact of disability has been somewhat neglected in relation to education (Bines & Lei 2011). For instance, United Nations Educational, Scientific and Cultural Organization (UNESCO) estimates that 90% of children with disabilities in the global south do not attend schools with fewer than 10% of children with disabilities in Africa attending school (UNESCO 2020). In most African countries, children with disabilities persistently face barriers to education (Ben-David & Nel 2013). Although access to education ‘is a fundamental human right, children with disabilities in sub-Saharan Africa are often excluded and marginalised’ (Deluca, Tramontano & Kett 2014; Dube et al. 2021:1; United Nations n.d.).

In 2012, it was estimated that approximately 600 000 learners with disabilities were not in school (Department of Basic Education [DBE] 2015), which is more than double the 280 000 estimated excluded learners in 2001 (DoE 2001). The 2011 census indicates that persons with severe disabilities are the most disadvantaged when it comes to educational outcomes (Statistics South Africa [SSA] 2011). This exclusion from education is out of line with the Constitution of the Republic of South Africa (Act no. 108 of 1996) and the goals of Education White Paper 6 (EWP6) (DoE 2001). Furthermore, even for those learners who are in school, their learning and participation are not at all satisfactory (Kelly & McKenzie 2018), with only 0.5% of all learners writing the National Senior Certificate in 2018 being recorded by the DBE as having special educational needs (DBE 2018). The national prevalence rate of disability amongst school-age children is between 2.6% and 10.8% (SSA 2011).

The Including Disability in Education in Africa (IDEA) Research Unit at the University of Cape Town has evolved from the university’s Teacher Empowerment for Disability Inclusion (TEDI) project, which was created in response to a call to address this exclusion and poor-quality education of children with disabilities in South Africa (http://www.dhrs.uct.ac.za/dhrs/divisions/disability/tedi). As commented by Cosier and Pearson (2016), the fields of Disability Studies and teacher education ‘have not communicated and collaborated in deep and meaningful ways’ (p. 1) in the past. Communication and collaboration between these two parties could result in a more inclusive and higher quality education for children with disabilities in South Africa.

The TEDI project was, therefore, developed by the Disability Studies Division in the Department of Health and Rehabilitation Sciences at the University of Cape Town in partnership with Christoffel-Blinden Mission (CBM) and co-funded by the European Union and CBM to address the exclusion and poor-quality education of children with disabilities in South Africa (http://www.dhrs.uct.ac.za/dhrs/divisions/disability/tedi). In order to provide an empirical basis for our work, we conducted research studies resulting in the following reports:

- Teacher education: An analysis of the availability of teacher education addressing the educational needs of learners with severe to profound sensory or intellectual impairments
- Starting where we are: Situational analysis of the educational needs of learners with severe to profound sensory or intellectual impairments in South Africa
- Perceptions of South African teachers on how they feel supported in teaching learners with special educational needs: Perspectives on inclusive education in South Africa
- Educating and caring for children with profound intellectual disability: A manual for carers and teachers.

Drawing on the above-mentioned research into learner and teacher education needs, 5-day face-to-face courses were developed for each of these focus areas. A total of 114 South African educators have participated in the face-to-face courses nationally. The four courses were on:

- disability studies in education
- the education and care of learners with severe to profound intellectual disabilities
- teaching learners who are blind or have low-vision
- teaching learners who are deaf or hard of hearing.

Complementary 4–5-week massive open online courses (MOOCs) were also developed. Over 8000 people have participated in the MOOCs, to date. In the process, we have learned a great deal about disability inclusion in education.

In addition, TEDI has developed a network of stakeholders in the education of children with severe to profound disabilities with government departments, civil society and institutions of higher education.

The TEDI project came to an end in August 2020 and evolved into a new research unit IDEA at the University of Cape Town.

Within the South African inclusive education policy, the move away from educational provision on the basis of disability category towards meeting identified support needs has rendered any reference to disability as problematic and even counter to the broad vision of inclusive education, given the negative effects associated with labelling practices (Baglieri & Shapiro 2017). However, a distinction between different types of barriers remains within policy between those barriers that are extrinsic to the child (e.g. social and curriculum barriers) and those that are intrinsic to the child (impairment related) (McKenzie et al. 2020; Walton et al. 2009).

We adopt a disability studies perspective, which blurs the distinction between intrinsic and extrinsic barriers because any impairment can be more or less disabling depending on the social conditions existing in the environment. Impairments are viewed as an interaction between a person and their environment and not as purely intrinsic to the child (Baglieri et al. 2011). We are aligned to a disability studies in education (DSE) approach, which contextualises disability as a political and social phenomenon and foregrounds the experiences and voice of disabled people and their families. Disability studies in education seeks to promote social justice and equitable educational opportunity and rejects a deficit model of disability preferring to focus on asset-based approaches such as universal design for learning (UDL) (Baglieri et al. 2011; Connor 2019).

This approach resonates with the International Classification of Functioning, Disability and Health (World Health Organization 2002), where disability is seen in the light of how the functioning and disability of an individual occurs in a context and includes a list of environmental factors. We therefore unashamedly use the term disability reinstating the disability label within inclusive education as a form of resistance to the neglect of impairment-specific needs within the system (McKenzie, Kelly & Shanda 2018). In so doing, we do not abandon the notion of barriers to learning and view these barriers as impacting upon learners with and without disabilities and accepting that inclusive education is about addressing all barriers to learning but that specific barriers might require specific types of support.

Recognising that disability is a significant (but by no means the only barrier), the Research Unit is to hone in on disability as a significant barrier ‘at both intrinsic and extrinsic levels in the parlance of South African education policy’ (McKenzie et al. 2020:4) within an inclusive education framework, paying attention to both disability studies and education policy.

### The Including Disability in Education in Africa vision

- To promote the inclusion of disability in education at all levels, both formal and informal, in Africa and beyond, to ensure no-one is left behind in the pursuit of equitable quality education and lifelong learning

### The Including Disability in Education in Africa mission statement

- To provide expert, relevant and comprehensive research on disability inclusion in education in Africa, by focusing on the education and support of people with disabilities, their families and their communities within the context of inclusive educational systems
- To facilitate the development of appropriate and relevant curriculum frameworks for disability inclusion
- To develop and disseminate innovative face-to-face and online training in inclusive education for teachers, education officials, support workers, community stakeholders, therapists and others
- To conduct multifaceted research pertinent to policy development and implementation in inclusive education and explore the barriers and supports that people with disabilities experience in accessing meaningful education
- To stimulate dialogue and discussion regarding disability inclusion amongst all relevant stakeholders and networks

### The Including Disability in Education in Africa primary objective

- To act as hub for future research in inclusive education in Africa and beyond, to promote networking and to carry out training within this field. It is envisaged that the unit will be a catalyst to further ideas and knowledge, and promote and strengthen the area of inclusive education locally, nationally, regionally and globally.

### The work of Including Disability in Education in Africa

The IDEA Research Unit operates in the areas of research, networking and training services:

## Research

The IDEA Research Unit’s main focus is its research in disability inclusion in education in Africa with the aim of providing quality, applied, action and impact research. This research will thus address the current paucity of knowledge in this particular area of education and will subsequently inform and support decisions that need to be made to make education more inclusive. The overall objective of IDEA is to provide expert, relevant and comprehensive research on disability inclusion in education in Africa, paying specific attention to the education and support of children with disabilities and their caregivers, families and communities within the context of inclusive educational systems.

We are dedicated to the provision of expert, relevant (culturally congruent) and comprehensive research and consultation and multifaceted approaches and/or multi-stakeholder collaborative practices for persons with disabilities, caregivers, families and communities within the context of quality, inclusive educational systems.

Through IDEA, we will facilitate the development of appropriate and relevant curriculum frameworks for disability inclusion and conduct multifaceted research pertinent to policy development and implementation in inclusive education.

The core research areas are as follows:

- analysis of data already collected by TEDI as it relates to teacher empowerment and disability inclusion
- evaluation of the face-to-face and online training courses developed by TEDI
- an investigation into the ways in which inclusive education policy and practice in South Africa enables quality and equitable education for children with disabilities
- comparative studies of inclusive education in the Global South
- exploration of instructional and social practices that support inclusion, such as teacher education, parent support, leadership skills and similar studies
- theoretical perspectives on disability in education, drawing upon frameworks of critical disability studies and post-colonial theory.

## Networking

The focus of the part of the research unit is to network with other universities, research organisations, non profit organisations (NPOs) disabled persons organisations (DPO) and other relevant stakeholders in the field of inclusive education. Through networking, IDEA also seeks to advocate for the right to equitable and inclusive education for learners with disabilities, highlighting the need for stakeholders at all levels to view the vision of IDEA as a human rights issue. Education is a human right for all children, which was affirmed by the Universal Declaration of Human Rights (United Nations n.d.). The network will facilitate collaboration that will strengthen advocacy and programmes around disability and education. Relationship with these stakeholders is fundamental to building a unified stance in the approach to developing a more inclusive education.

Networking plays an integral role in building and further developing the landscape of teacher education for inclusive education. Through IDEA we aim to stimulate dialogue and discussion regarding disability inclusion amongst all relevant stakeholders and networks. We also aim to contribute to scientific knowledge in the field of disability inclusion in education, broaden awareness and showcase the importance of disability inclusion in education.

We act as the secretariat for the Association for the Advancement of Inclusive and Equitable Education in South Africa (AAIEESA).

Networking is intrinsically linked to capacity building as the sharing of knowledge and experiences across different learning communities empowers community groups to participate in inclusive education in their own communities. To this end, we have resources for parents and teachers on our webpage and we run free webinars on different aspects of disability and inclusive education.

## Training

Training is also a key focus of the unit. Training and teaching of educators, parents, communities and other stakeholders on how to be more comprehensive in their respective work is imperative to make inclusive education more accessible and participative at grassroots level. It is only through training that stakeholders will be able to be more effective and impactful to children, scholars and students with disabilities. The training offered through IDEA also emphasises the need for the various role players in the inclusive education sector to build communities of practice and gain professional agency. A service that IDEA provides is the development and dissemination of innovative face-to-face and online training in inclusive education for teachers, education officials, support workers, therapists and other inclusive education stakeholders. The online and face-to-face courses are focused on the following two areas of need:

- training for teachers and district officials
- training for higher education institutions, especially technical vocational education and training (TVET) and private teacher education colleges.

Examples of the work we do:

One of the IDEA Research Unit’s first research projects was to perform an evaluation study for CBM-International (CBM-I).

Christoffel-Blinden Mission-International approached the IDEA Research Unit requesting it to perform consultancy on a programme evaluation aimed at assessing the effectiveness of its Community Based Inclusive Development (CBID) partner driven projects. These projects have a variety of specific aims, but broadly support disability-inclusive development and the creation of social and material environments, which promote the participation of people with disabilities in all aspects of community life.

The evaluation will focus on collecting baseline data (in 2021) and follow-up data (in 2022 and 2024) from 16 programmes across eight countries (Zimbabwe, Rwanda, Ethiopia, Togo, Cameroon, India, Honduras and Pakistan), all with diverse goals in inclusive development.

The following were objectives of the evaluation:

- To reflect an accurate picture of the status quo in selected CBID programmes in eight countries in relation to the objectives provided in CBM’s own CBID initiative plan
- Enrich an understanding of the communities in which CBM-I’s projects operate, including the identifying of problem areas, in order to facilitate improvement in the implementation of the ideals of CBID
- Including Disability in Education in Africa was also commissioned by CBM to undertake a review to identify current UDL practices, training needs and relevant online resources in low- and middle-income countries (LMICs). The specific terms of reference were to review the current practice of UDL in LMIC settings with a view to forming recommendations for capacity development resources and materials, including:
- Literature review of the use of UDL in LMIC
- Exploration of the potential of UDL to address systemic discrimination based on race or ethnicity and disability
- Recording the online UDL materials that are available into a database
- Interviewing several key informants
- Making recommendations for online learning for UDL in LMICs
- A report titled *Review of Universal Design for Learning in low- and middle-income countries* was written and submitted to CBM (McKenzie et al. 2021).

## Conclusion

The IDEA Research Unit was established in recognition that there was a gap in the inclusive education arena – especially in the global south – and so it created a more focused approach to challenge this state of affairs. With more relevant research, networking and training, IDEA hopes to highlight the issues pertaining to disability and education from a critical perspective that interrogates the application of globalised concepts and theories in the African context. In so doing, African approaches and strategies to addressing barriers to learning and development will be explored, making a contribution to the international literature on inclusive education.
